# Novel mutations in GJB1 trigger intracellular aggregation and stress granule formation in X-linked Charcot-Marie-Tooth Disease

**DOI:** 10.3389/fnins.2022.972288

**Published:** 2022-09-26

**Authors:** Fan Chu, Jiaming Xu, Yong Wang, Yingjie Li, Yaling Wang, Zhijun Liu, Chuanzhou Li

**Affiliations:** ^1^Department of Medical Genetics, School of Basic Medicine, Tongji Medical College, Huazhong University of Science and Technology, Wuhan, China; ^2^Department of Neurology, Union Hospital, Tongji Medical College, Huazhong University of Science and Technology, Wuhan, China

**Keywords:** GJB1, gap junction, aggregation, stress granule, Charcot-Marie-Tooth

## Abstract

X-linked Charcot-Marie-Tooth Disease type 1(CMT1X) is the second most common form of inherited peripheral neuropathy that is caused by mutations in the gap junction beta-1 (GJB1) gene. Using targeted exome-sequencing, we investigated four CMT families from central-southern China and identified two novel missense variants (p.F31S and p.W44G) and two previously reported variants (p.R220Pfs^*^23 and p.Y157H) of GJB1. All four probands presented typical early-onset peripheral neuropathy, of which the R220Pfs^*^23 carrier also had neurologic manifestations in the central nervous system. We then constructed GJB1 expression vectors and performed cell biological analysis *in vitro*. Expression of FLAG-tagged GJB1 at various time points after transfection revealed evident protein aggregation with both wild-type and mutant forms, indicated with immunostaining and immunoblotting. Detergent-based sequential fractionation confirmed that all mutants were higher expressed and more prone to aggregate than the wild-type, whereas the R220Pfs^*^23 mutant showed the greatest amount of SDS-soluble multimers and monomers among groups. Moreover, intracellular aggregation probably occurs in the endoplasmic reticulum compartment rather than the Golgi apparatus. Gap junction plaques were present in all groups and were only compromised in frameshift mutant. Further evidence reveals significant intracellular stress granule formation induced by mutated GJB1 and impaired cell viability indicative of cytotoxicity of self-aggregates. Together, our findings demonstrate novel GJB1 variants-induced cell stress and dysfunction and provide insights into understanding the pathomechanisms of GJB1-CMTX1 and other related disorders.

## Introduction

Charcot-Marie-Tooth (CMT) disease is a genetically heterogeneous group of disorders characterized by progressive distal muscle weakness and atrophy, usually with foot deformities and sensory loss, as well as decreased tendon reflexes (Szigeti and Lupski, 2009). CMT usually starts in the first or second decade of life, although it may also affect infants or individuals with advanced age. CMT can be divided into autosomal dominant, autosomal recessive, and X-linked dominant or recessive types according to the mode of inheritance. The X-linked dominant form of CMT type 1 (CMT1X), usually caused by mutations in the gap junction beta-1 (*GJB1*) gene, is the second most common form of inherited peripheral neuropathy and accounts for approximate 10% of all CMT cases (Fridman et al., 2015).

*GJB1-*encoded GJB1 protein, also named connexin-32 (Cx32), is widely expressed in peripheral myelin and is specifically located at uncompacted folds of Schwann cell cytoplasm around the nodes of Ranvier and Schmidt-Lanterman incisures (Abrams and Freidin, 2015). It is also found in the outer oligodendrocyte membranes in the central nervous. Individuals with GJB1-related CMT1X usually not only manifest as peripheral nerve impairment but also have concurrent involvement of the central nervous system (CNS), such as stroke-like episodes, dysarthria, spasticity, ataxia, and cognitive impairment (Caramins et al., 2013; Xie et al., 2016; Abrams et al., 2017). Like other X-linked dominant disorders, GJB1-CMT usually affects males more severely than females, some of whom may have mild symptoms or remain asymptomatic.

More than 450 different *GJB1* mutations associated with CMT1X have been identified, which affect the protein-coding region as well as the non-coding region of *GJB1* (Tomaselli et al., 2017; Liu et al., 2020). These include missense, nonsense, frameshift, deletion, as well as splicing mutations (http://molgen-uia.ac.be/CMTMutations/). Mutations in *GJB1* are widely distributed across the whole gene, although the extracellular-2 (EC2) domain, the first transmembrane domain 1, and the distal C-terminus are considered as possible mutational hotspots in Chinese CMT1X patients (Lu et al., 2017). Most *GJB1* mutations lead to partial or complete loss of GJB1 function, which results in abnormalities in protein intracellular trafficking, protein expression, and gap junction plaque formation (Kleopa et al., 2012). There is also evidence that several *GJB1* variants may also have toxic gain-of-function effects on the CNS (Olympiou et al., 2016).

In this study, we conducted a targeted exome-sequencing in CMT families from the central-southern region of China and revealed several novel *GJB1* mutations. We demonstrated that these mutants altered GJB1 expression and augmented properties prone to constitute self-organized aggregates or insoluble fibrils mostly in the endoplasmic reticulum compartments. Further evidence indicates that mutated GJB1 induces significant intracellular stress granules (SGs) formation and impaired cell proliferation indicative of cytotoxicity. Our findings disclose that novel GJB1-CMT mutations cause impaired structure organization of GJB1 and deposition of accumulated aggregated GJB1 species, resulting in a boost of cell stress and depletion of cell viability in CMT pathology.

## Materials and methods

### Patients

A total of 10 patients from four CMT families, including four probands and six affected family members, were recruited for this study. All affected patients were evaluated by at least two neurologists and diagnosed according to the criteria recommended by the European CMT Collaborative Research Group. All patients were of Han Chinese origin, were from the central-southern region of China, and were recruited at the Neurology Department of Wuhan Union hospital, between January 2018 and September 2021. Genomic DNA was extracted from peripheral blood samples obtained from all affected individuals and their family members. Written informed consents were obtained from all participants. This study was approved by the Ethics Committee of Wuhan Union Hospital, and all human participants' relevant procedures were performed in accordance with the Declaration of Helsinki.

### Genetic analysis

Genomic DNA was extracted from peripheral blood samples using a standard DNA extraction method. Deletion/duplication analysis on the PMP22 gene was performed by Multiplex ligation-dependent probe amplification assay (MLPA) as reported previously (Slater et al., 2004). A target panel, comprising 119 genes currently known to be involved in CMT and other associated hereditary neuropathies, was designed to perform comprehensive genomic testing. Deep sequencing was performed using the Illumina Hiseq2000 system. The annotation and analysis of sequenced reads were performed as we described previously (Liu et al., 2019). Filtering of all variants was performed using all variants from publicly available databases including the 1000 Genomes Project, the Exome Sequencing Project 6500 (ESP6500), and the Exome Aggregation Consortium (ExAC) database. The probable effects of variants on protein function were predicted using SIFT, PolyPhen2, and Mutation Taster. Sanger sequencing was used to validate the candidate variants after data analysis. Co-segregation analysis was conducted through screening for the confirmed variants in the family members.

### Construction GJBI-expressing vector and mutagenesis

The cDNA clone plasmid containing human GJB1 (NM_001097642) was purchased from Youbio Biotechnology (Cat No. G109013, Changsha, China). cDNA fragment was then sub-cloned into eukaryotic expressing vector pEGFP-N1 using EcoR1 and BamH1 restriction sites. F31S and W44G mutations were accomplished using the QuickMutation kit (Beyotime Biotechnology, D0206), according to the manufacturer's instructions. Wild-type and mutated fragments of *GJB1* were sub-cloned into pcDNA3.1-FLAG vector using EcoR1 and XhoI restriction sites. For R220Pfs^*^23 mutation, c.657dupC mutation was achieved using a mutagenesis kit, and the resultant truncate coding sequence of *GJB1* was then sub-cloned into pcDNA3.1-FLAG vector to ensure in-frame expression of FLAG tag at the c-terminus. Sequences of all constructs were validated by Sanger sequencing.

### Cell culture and plasmid transfection

The human cervical cancer HeLa cell line was obtained from China Center for type culture collection (CCTCC). Cells were routinely maintained in Dulbecco's modified eagle medium at 37°C (5% CO_2_) (Gibico). The medium was supplemented with 10% fetal calf serum (Life Technologies) and 1% penicillin-streptomycin (10,000 U/ml, Life Technologies). Cells were seeded in 12-well plates or Petri dishes for immunofluorescence and western blotting analysis. Cells were plated onto plates for 18–24 h before transfection at a confluence of 60% to 70% or onto coverslips as required. Previously described plasmids and control vectors were prepared and quantified. Transfection was conducted using a polyethylenimine reagent (PEI, Polyscience). In brief, for each well in a 12-well/plate, 1 μg DNA was diluted in 200 μl Opti-MEM (Gibco) followed by mixing with 1 μl PEI solution (5 mg/ml). The transfection mixture was incubated at room temperature for 20 min before being added to each well. Cells were harvested 24 h post-transfection for immunoprecipitation, immunostaining, and western blotting assays as needed.

### Cell counting kit-8 assay

HeLa cell suspension was inoculated at 15,000 cells per well in a 96-well plate, which was later pre-incubated for 24 h in a humidified incubator at 37°C, 5.0% CO2 with saturated humidity. For each well, a 10 μl transfection mixture including 200 ng of total DNA and 0.5 μg PEI was incubated and added into the cell medium, followed by 24 h in the incubator. Subsequently, 10 μl of CCK-8 solution (Biosharp Life Science, BS350A) was added to each well of the plate. Bubbles were avoided when reading absorbance. Plates were incubated for 1 h before absorbance measurement at 450 nm.

### Western blotting and sequential extraction

For most immunoblotting assays, cells were collected and lysed in a 2 × Laemmli sample buffer containing SDS (Bio-Rad). They were then thoroughly lyzed by sonication and heat denatured at 95°C for 5 min before loading onto gels for analysis. For the non-heat-denatured condition, samples were lysed in the same buffer as above followed by sonication and were loaded onto gels without the heat-denaturation step. For sequential extraction, cells were processed as illustrated in the main text, and clear supernatant was collected and mixed with SDS-sample buffer before heat-denatured. Equal amounts of protein were loaded (10–50 μg depending on the assay) and resolved by SDS-PAGE in Tris-glycine-SDS buffer (Bio-Rad), followed by transfer onto nitrocellular membranes (Merck Millipore). The membranes were blocked with blocking buffer (Beyotime, P0256) for 0.5 h, and then reacted with primary antibodies in primary antibody dilution buffer (Beyotime, P0023A) overnight at 4°C under gentle rocking. Primary antibodies are as follows: anti-FLAG antibody (Beyotime biotechnology, AF519-1, 1:2,000 dilution), anti-ubiquitin antibody (Cell Signaling Technology, 3936P, 1:2,000 dilution), anti-GJB1 (Cx32) antibody (Santa Cruz Biotechnology, #10519, 1:2,000 dilution), and anti-GAPDH antibody (Abcam, AB-516, 1:4,000 dilution). After washing, the corresponding IR Dye 680RD/800CW secondary antibodies (LICOR, 1:10,000 dilution) were added for 1 h at room temperature under constant agitation. After washing with tris-buffered saline wash buffer with Tween 20, the membrane was scanned in the Odyssey Fc Imaging system (LICOR) for the detection of an infrared signal. Quantification of immunoblots was performed using ImageJ, by normalizing the band density of the target signal to internal control in each sample. Relative fold changes were presented after normalized to the average of the wild-type control group.

### Immunofluorescence staining

Cells grown on coverslips were washed gently with phosphate-buffered saline (PBS) and fixed in 4% PFA, followed by permeabilization with 0.3% Triton X-100 in PBS. The cells were then blocked for 1 h in 5% goat serum, followed by incubation overnight with primary antibodies at 4°C. Mouse anti-FLAG antibody (Beyotime biotechnology, AF519-1, 1:500 dilution), rabbit anti-FLAG antibody (Beyotime biotechnology, AF0036, 1:500 dilution), anti-GJB1 (Cx32) antibody (Santa Cruz Biotechnology,#10519, 1:150 dilution), anti-Calnexin antibody (Abcam, ab22595, 1:400 dilution), anti-GM130 antibody (Sigma-aldrich, 610822, 1:400 dilution), and anti-G3BP1 antibody (Beyotime biotechnology, AF0039, 1:150 dilution) were used. Secondary antibodies were Alexa Fluor (488, 555)-labeled goat anti-mouse and goat anti-rabbit antibodies (Thermo Fisher, 1:500 dilution). DAPI was used to visualize the nuclei. Fluorescence images were captured with a 20 × or 63 × objective on a Zeiss LSM780 laser scanning confocal microscope.

### Immunoprecipitation

Immunoprecipitation was performed following standard procedures. In brief, cells were washed in PBS and lysed in cold NP-40 buffer (50 mM Tris-HCl, pH 7.4, 150 mM NaCl, 2 mM EDTA, 1% NP-40) supplemented with a protease/phosphatase inhibitor cocktail (Cell Signaling Technology). The lysate was briefly sonicated and centrifuged at 12,000 rpm for 10 min at 4°C. The supernatant was then collected and 1 mg of total protein lysate was incubated with protein A-conjugated agarose beads (Beyotime technology, P2051) and primary antibody overnight at 4°C. Rabbit anti-FLAG antibody (Beyotime biotechnology, AF0036, 1: 200 dilution) was used. The beads were spun down, washed with PBS buffer, and denatured with 2 × Laemmli sample buffer (Bio-Rad), followed by western blotting for validation.

### Colocalization analysis and stress granule quantification

Colocalization of GJB1-FLAG and Calnexin or GM130 was conducted using Coloc 2 plugin function in ImageJ. Target regions in multichannel images were selected and two subject channels were split and properly thresholded. Channel images were selected in Coloc 2 for calculation of Pearson's *R*-value (above threshold). Immunofluorescent images after staining with anti-FLAG (GJB1-FLAG) and stress granule (SG) marker G3BP1 were captured at required channels and were merged for statistical analysis. Five fields with GJB1-FLAG expressing cells in all groups were thresholded identically to discriminate ectopic FLAG expression. SG-positive cells were characterized by the apparent appearance of a number of intense G3BP1-positive puncta in the cell instead of diffuse labeling across the cell. None of the SG-positive and FLAG-negative cells were observed. The number of FLAG-positive and SG-positive cells in individual images was finally counted and summed for the percentage of SG-positivity in GJB1-expressing cells.

### Statistical analysis

The biochemical analysis was performed using a minimum of three biological replicates per condition. The statistical analyses were done using the Prism 9 software (GraphPad). No data points were excluded from the analysis, and randomization of experimental groups was not required. Three or more groups were assessed with one-way ANOVA followed by multiple comparisons vs. the wild-type control as indicated. *P*-values close to significance were also shown. Values are presented as the mean ± standard error of the mean (s.e.m).

## Results

### Identification of *GJB1* variants by targeted exome-sequencing and sanger sequencing

**Figure 1:**
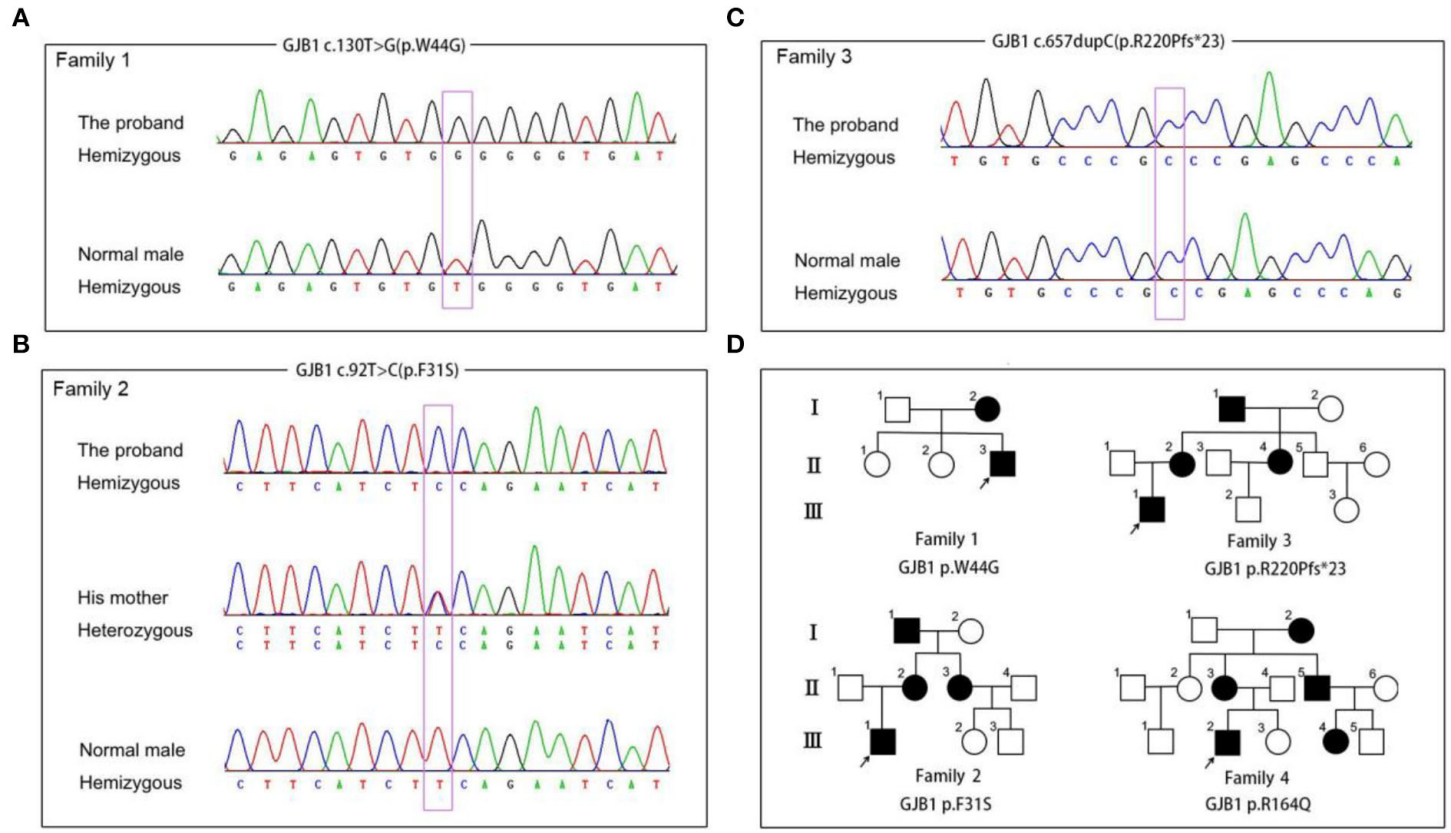
Segregation of GJB1 mutations. **(A)** Sequence chromatogram of the c.130T>G (p.W44G) missense mutation in family 1. **(B)** Sequence chromatogram of the c.92T>C (p.F31S) missense mutation in family 2. **(C)** Sequence chromatogram of the c.657dupC (p.R220pfs*23) frameshift mutation in family 3. **(D)** Pedigrees of families with GJB1 mutations in the study. Corresponding mutation sites are highlighted in purple boxes.

In general, about 97.3% of the target bases were covered with at least 20 × per individual, and the mean depth of coverage for all target regions was 112. After filtering and validation by Sanger sequencing, four probable pathogenic variants, including two variants previously reported as pathogenic variants (c.469T>C, p.Y157H, (Li et al., 2016) and c.657dupC, p.R220Pfs^*^23) (Lu et al., 2017) and two novel variants (c.130T>C, p.F31S and c.92T>C, p.W44G), were identified in four CMT families (Figure 1). The two novel variants, being predicted as harmful effects by the SIFT or PolyPhen-2 software, were absent in publicly accessible databases (the 1,000 Genomes Project, ExAC, and ESP6500 databases). In addition, some missense variants (p.W44C and p.W44L) affecting the same amino acid sequence of GJB1 were previously reported as pathogenic variants (Bone et al., 1997; Sharkova et al., 2012). According to the American College of Medical Genetics and Genomics (ACMG) standards and guidelines, all these variants were classified as likely pathogenic variants.

### Clinical manifestations of CMT patients

The clinical and electrophysiological characteristics of all probands are summarized in Table 1. All probands were male patients and there was no expected instance of male-to-male transmission in any of the pedigrees. All patients had a typical manifestation of peripheral neuropathy, including early age-at-onset, progressive distal muscle weakness and atrophy, decreased tendon reflex, and high foot arch. The clinical manifestations of patients with *GJB1* mutation are described in detail as follows.

**Table 1:** The clinical presentations and genetic analysis of four CMT probands.

**Characteristics**	**Proband 1**	**Proband 2**	**Proband 3**	**Proband 4**
Age, years	29	23	25	28
Sex	Male	Male	Male	Male
Age at onset, years	23	22	Early childhood	15
Initial symptoms	Weakness and atrophy in hands	Weakness of right hands	Running difficulty	Right lower limbs weakness
Pathogenic variants in *GJB1*	p.W44G	p.F31S	p.R220Pfs^*^23	p.Y157H
Muscle atrophy UL/LL	+/++	++/+	++/++	++/+++
Deep tendon reflexes	Decreased	Decreased	Disappeared	Disappeared
Pes cavus	Yes	Yes	Yes	Yes
Sensory loss	No	Yes	No	Yes
Brain MRI	Normal	Normal	Hyperintensity in the bilateral corona radiate on T2WI	Normal
Other features	–	–	Stroke-like episodes	–
CMAP in median/ulnar nerves, mv	1.0/3.5	0.3/0.7	1.0/3.1	NA
MCV in median/ulnar nerves, m/s	29.9/32.4	25.3/26.8	28.6/31.1	NA
SNAP in median/ulnar nerves, uv	5.5/7.0	4.0/4.5	4.6/6.3	NA
SCV in median/ulnar nerves, m/s	38.2/37.3	32.1/33.4	35.4/36.5	NA

UL, upper limbs; LL, low limbs; +, mild; ++, moderate; +++, severe; CMAP, compound motor action potential; MCV, motor conduction velocity; SNAP, sensory nerve action potential; SCV, sensory conduction velocity; NA, not available.

The proband 1 (II:3) was a 29-year-old male carrying the p.W44G *GJB1* variant. He had weakness and atrophy in his hands at the age of 23 years and presented lower limb weakness at the age of 26 years. His examination was further marked by decreased deep tendon reflexes, pes cavus, and mild weakness and atrophy in all extremities, especially in the lower limbs. He received orthopedic surgery because of severe pes cavus deformity at the age of 28. Brain magnetic resonance imaging (MRI) was normal. Electrophysiological findings showed axonal degeneration and demyelinating polyneuropathy with slowing motor nerve conduction velocity and reduction in amplitude. His mother (I:2) carrying a heterozygous mutation presented with delayed walking in childhood, and was wheelchair-bound for 20 years after symptom onset. While no signs or symptoms of CMT were observed in his two elder sisters.

The proband (III:1) in family 2, carrying the p.F31S variant in *GJB1*, was a 23-year-old male who presented with weakness in right hands at the age of 22. As the disease progressed, muscle weakness and atrophy soon spread to affect the distal muscles of the upper and lower limbs. He also had occasional numbness around his hands. No signs of CNS involvement, including intellectual disability and dysarthria, were observed in the proband. Neurological examination showed prominent weakness and atrophy of the small muscles of the hands, mild to moderate weakness and atrophy of distal lower limbs, and bilateral pes cavus. The deep tendon reflexes were decreased in both upper and lower extremities except for biceps and triceps reflexes. Electrophysiological results showed reduced motor and sensory nerve conduction velocity in the median, ulnar, and common fibular nerves, with a mild decrease in amplitude. MRI of the brain was unremarkable. The proband's mother (II:2) and aunt (II:3) were both heterozygous for this mutation and presented similar symptoms in their 30s.

The proband 3 (III:1) was a 25-year-old male, carrying the p.R220Pfs^*^23 variant in *GJB1*, who had noticed running difficulty since early childhood. As the disease progressed, he began to experience delayed walking and frequent falls. At the age of 18, he suddenly experienced left limb weakness and speech difficulties, and the symptoms completely recurred within 1 h. Over the last 5 years, he presented with weakness and atrophy of the distal muscles of both upper and lower limbs. Physical examination revealed mild weakness and atrophy in all extremities, disappeared deep tendon reflexes, and pes cavus. Electromyography (EMG) results showed abnormalities in axonal and demyelinating polyneuropathies involving both motor and sensory nerves. Brain MRI showed bilateral symmetrical corona radiata signal abnormality without any significant diffusion restriction. His mother (II:2), aunt (II:4), and maternal grandfather (I:1) were also affected with the same phenotype of leg weakness and high-arched feet before the age of 20. His mother (II:2) and aunt (II:4) were found to be heterozygous carriers of this mutation, while the maternal grandfather's DNA sample is not available for testing. No evidence of CNS involvement including episodic limb weakness or dysphasia was observed in other affected individuals within this family.

The proband 4 (III:2) was a 28-year-old male, carrying the p.Y157H variant in *GJB1*, who presented with right lower limb weakness at the age of 15. As the disease progresses, he experienced weakness and loss of sensation in his hands. Neurological examination revealed distal muscle weakness in all extremities, severe calf muscle atrophy, disappeared deep tendon reflexes, foot drop, and pes cavus. Other patients in the family (I:2, II:3, II:5, and III:4) experienced similar symptoms around the same age period as the proband. The EMG results showed typical features of demyelinating and axonal polyneuropathies. The brain MRI revealed no significant abnormality.

### Expression of novel *GJB1* variants in mammalian cells

**Figure 2:**
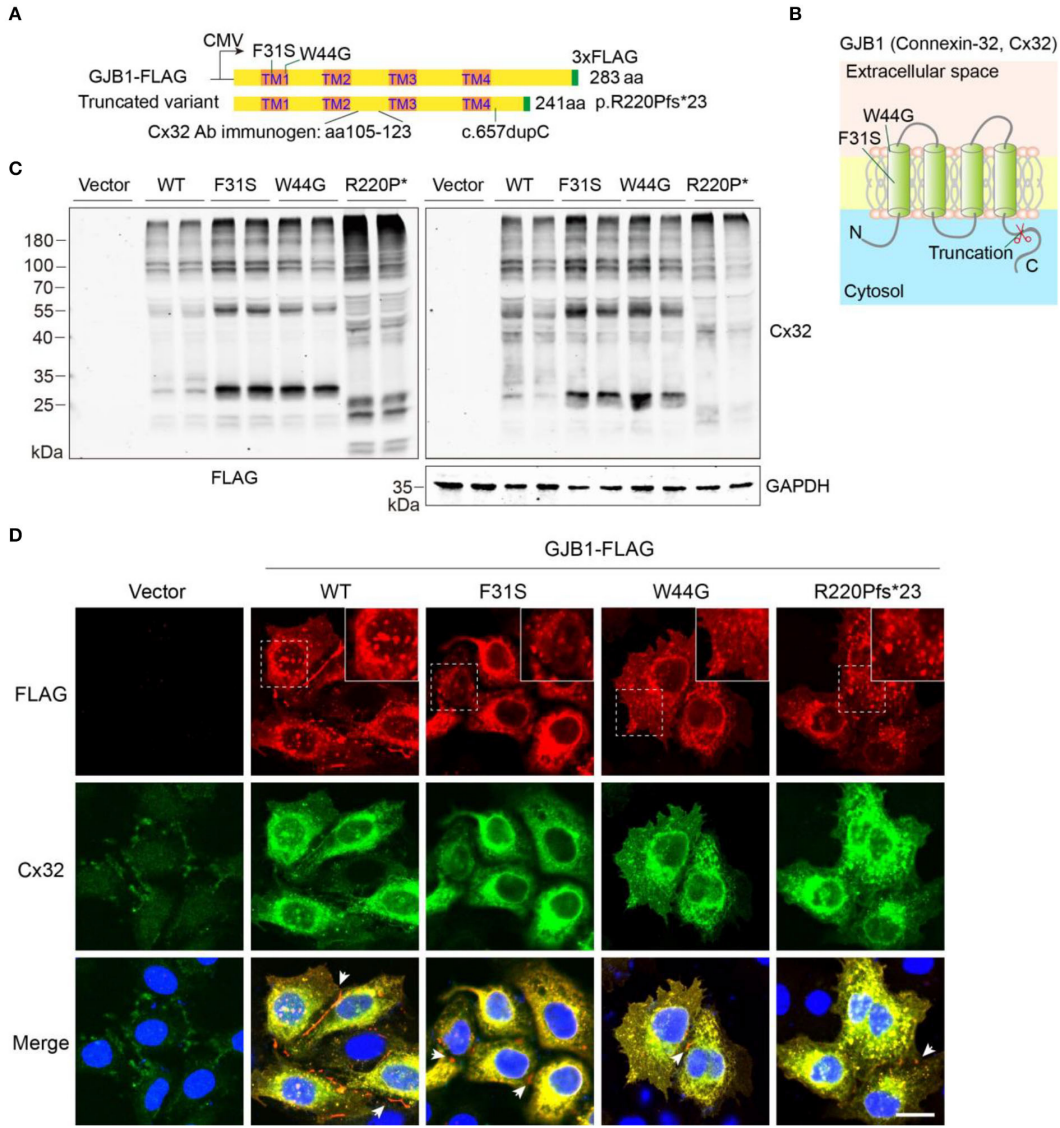
Eukaryotic expression of GJB1 variants. **(A)** Schematic illustration of vector design and domain structure of GJB1. TM1-4 represents transmembrane (TM) helical. CMV promoter was used. Mutation sites region of the immunogen for Cx32 antibody are indicated. **(B)** Model of GJB1 assembly on the plasma membrane with four TM domains. **(C)** Immunoblots of wild-type (WT) and mutant forms of GJB1 expression in HeLa cell lysates with anti-FLAG and anti-Cx32 antibodies. Cell lysates were collected 24 h after transfection. GAPDH acts as a loading control. R220P* represents truncated mutant R220Pfs*23. **(D)** Immunofluorescence staining of fixed HeLa cells with indicated transfection. Blue, DAPI-labeled nuclei. Enlarged views were shown at the top right corner. Arrowheads denote GJ-plaque structures that were preferentially labeled with anti-FLAG antibody. Scale bar, 20 μm.

To determine the functional consequence of the *GJB1* mutations, we performed a biochemical analysis *in vitro* by transfecting GJB1-expression vectors in HeLa cells and lysing the cells in a Laemmli sample buffer containing SDS to obtain total lysate (Figure 2A). Since functional alternation by Y157H variation has been briefly explored previously (Li et al., 2016), we have not included this mutation in our study. As shown, F31S and W44G mutations locate in the 1st transmembrane (TM) domain of GJB1, while R220Pfs^*^23 occurs in the C-terminal domain resulting in truncation in the cytosol (Figure 2B). Probing ectopic GJB1 expression in total protein extracts using anti-FLAG antibody showed no bands in the control vector (pcDNA3.1) transfected cells as expected. In the wild-type (WT) group, a 32-kDa band at the predicted size of full-length GJB1 was observed, as well as a lower band (~28 kDa) at a similar density. In the F31S and W44G groups, the 28-kDa band was the main band, while the main band in the truncated mutation was even lower as expected, with an extra band below. Interestingly, in all GJB1 transfected groups, besides the main bands ranging from 20 to 35 kDa, higher bands were apparently visible at the size of multimers as we suspected (Figure 2C). Overall, the FLAG and Cx32 antibodies detected similar signals, with the FLAG antibody preferentially detecting stronger signal than the Cx32 antibody with all species of truncated mutation, possibly due to the immunogen of Cx32 antibody located between TM2 and TM3 of GJB1 (Figure 2A). The immunoblots against FLAG suggested that GJB1 mutations present a higher amount of monomers and multimers.

We then confirmed whether all the GJB1 variants form aggregates intracellularly using immunofluorescent staining. As shown in Figure 2D, two antibodies showed highly overlapped signals confirming the recognition of GJB1-FLAG by the Cx32 antibody. Although expressed at a low level, endogenous GJB1 in the vector group was localized at intercellular junctions. As expected, ectopic expression of WT-GJB1 and the variants showed similar intracellular signals of puncta-like condensates, in addition to gap junction (GJ)-plaques at gap junctions which were preferentially detected by anti-FLAG staining (Figure 2D). Taken together, overexpression of both WT-GJB1 and the mutants may induce formation of GJB1 multimers and even aggregates in mammalian cells.

### Low GJB1 expression induces intracellular aggregation

**Figure 3:**
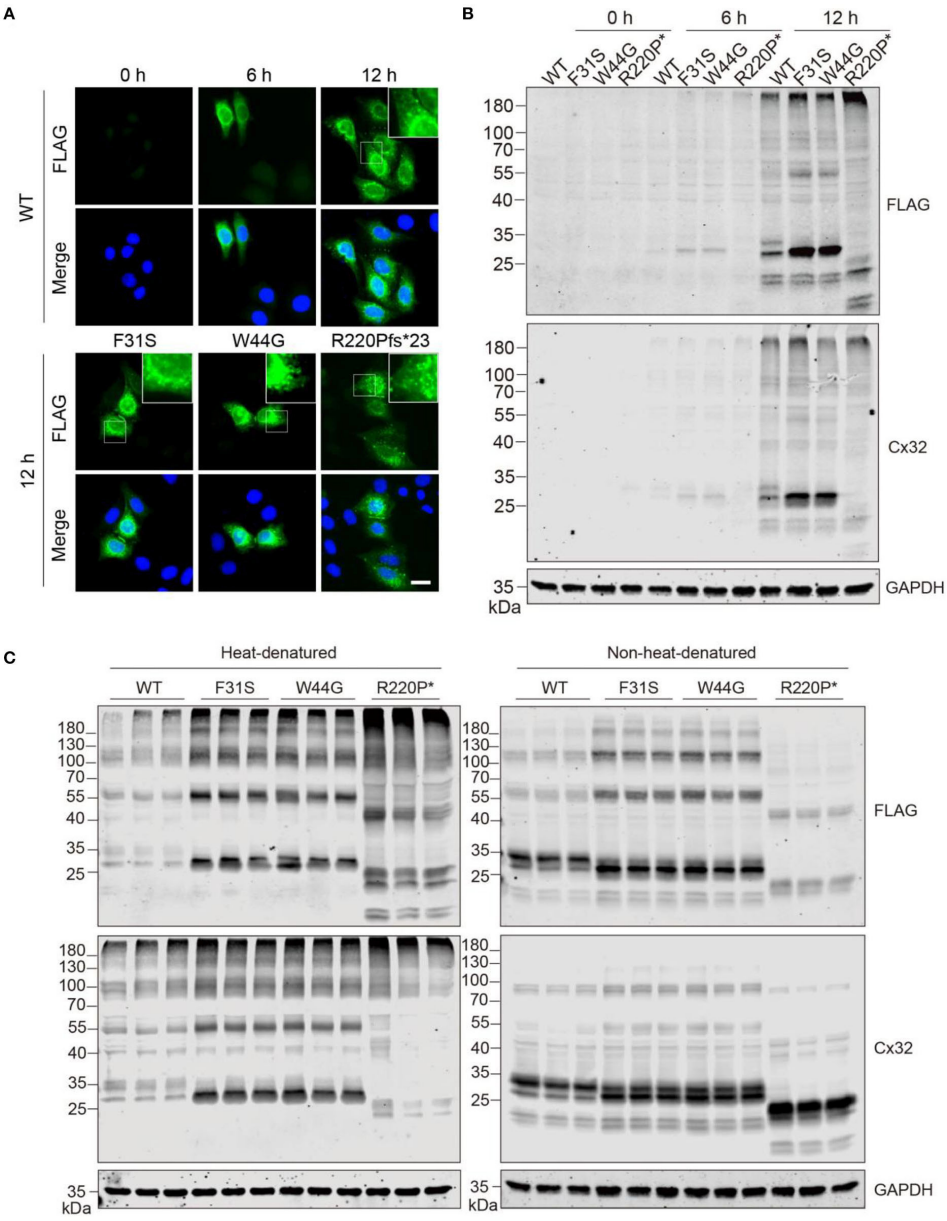
GJB1 forms puncta-like condensates in cells when expressed at a low level. **(A)** Immunofluorescence staining of GJB1 in overexpressing HeLa cells at indicated time points after transfection. Stainings of cells transfected with mutants at 12 h were shown. The top right boxes show an enlarged field. Blue, DAPI-labeled nuclei. Scale bar, 20 μm. **(B)** Immunoblots of GJB1 expression in HeLa cell lysate after indicated transfection. Anti-FLAG and anti-Cx32 antibodies were used. R220P* is short for R220Pfs*23. **(C)** Immunoblots of GJB1 expression in HeLa cell lysate 24 h after transfection. SDS sample buffer was used in the sample processing with or without heat-denaturation. Anti-FLAG and anti-Cx32 antibodies were used for detection.

Given transfection may sometimes cause an overload of overexpressed proteins that may lead to an aggregation effect, hence we attempted to analyze if aggregates will be formed when protein expression is low at an early stage after transfection. Puncta-like aggregates were consistently found at 6 and 12 h after expression in the WT group, similar to aggregates in the other groups (Figure 3A). Consistently, immunoblotting confirmed the observation by immunostaining that weak aggregation of GJB1 started to form 6 h post-transfection (Figure 3B), with similar pattern as detected at 24 h (Figure 2C). Since aggregation has been reported to be promoted by high temperature, especially when samples were boiled in the Laemmli buffer before electrophoresis, we compared in parallel when samples were heat-denatured or non-heat-denatured. SDS-PAGE of heat-denatured samples again replicated the patterns presented earlier, and non-heat-denatured lysate showed less amount of high-molecular-weight aggregates on top of the gels. However, a significant amount of multimers and monomers of GJB1 were present in both conditions independent of heat-denaturation. Intriguingly, the monomer band in the WT group was strongly positive than the F31S and W44G groups in which lower 28-kDa bands were the main band, and the WT monomer band was much reduced after heat-denaturation, suggesting the monomer full-length GJB1 was most affected by heat-denaturation (Figure 3C). When the samples were not boiled, the Cx32 antibody detects more GJB1 monomers with truncated mutation than the FLAG antibody but performed oppositely after boiling. Thus, the FLAG antibody might be more suitable for recognizing all species of GJB1 in classical heat-denaturation-based SDS-PAGE electrophoresis.

### Enhanced accumulation of insoluble GJB1 induced by mutations

**Figure 4:**
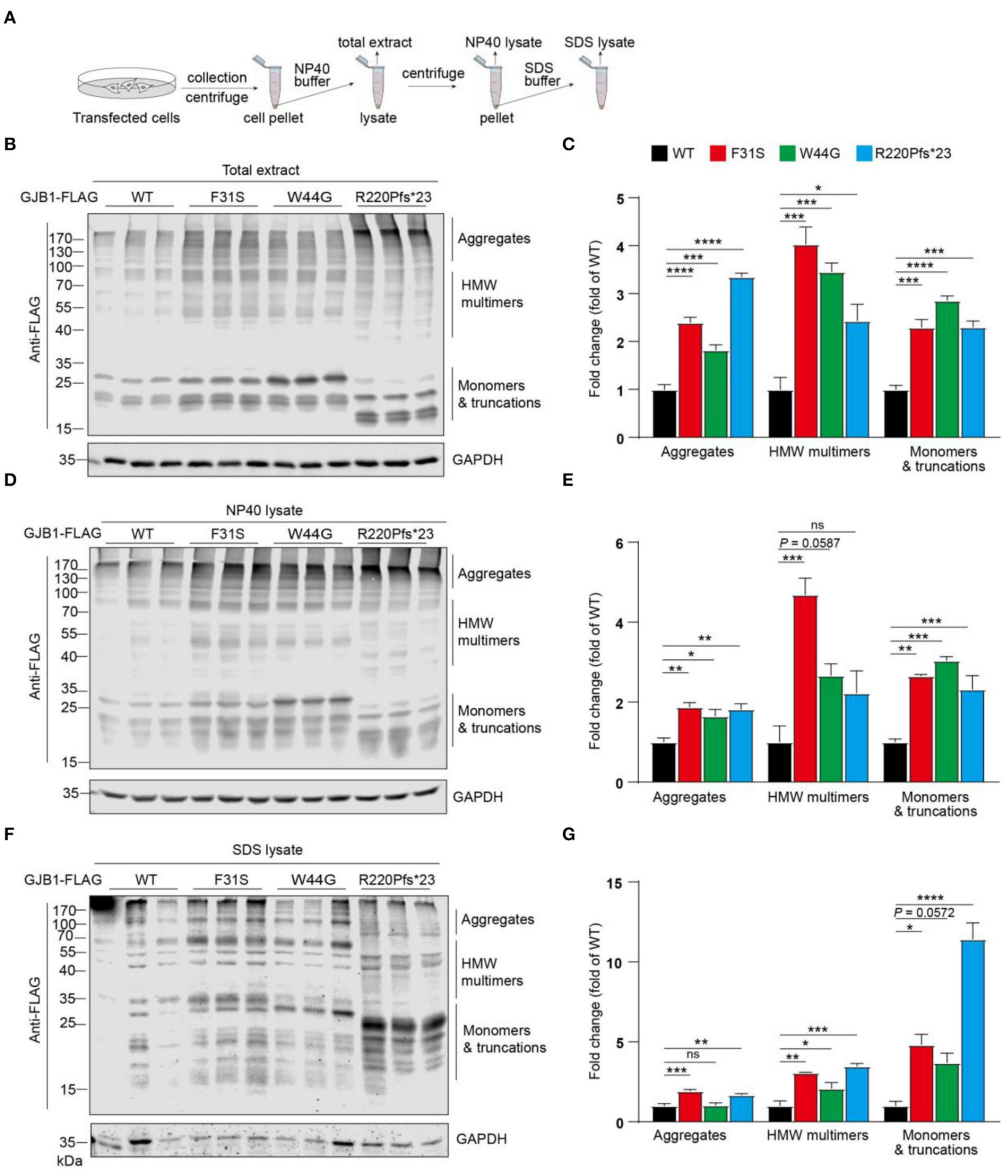
Sequential extraction analysis of GJB1 in overexpressing cells. **(A)** Illustration of stepwise extraction using NP40 buffer and SDS buffer to obtain a total extract, NP40 lysate, and SDS lysate. GJB1-transfected cell pellets were lysed in NP40 buffer for total extract collection. The supernatant was collected as N40 lysate after centrifugation. Pellet was then lysed and sonicated in SDS buffer to obtain SDS lysate. **(B)** Immunoblots of total extracts of cells expressing GJB1 variants using anti-FLAG antibody. HMW, high molecular weight. **(C)** Quantification of immunoblots in B. **(D)** Immunoblots of NP40 lysate of cells expressing GJB1 variants. **(E)** Quantification of immunoblots in D. **(F)** Immunoblots of SDS-lysate of cells expressing GJB1 variants. **(G)** Quantification of immunoblots in F. ns, not significant, *P < 0.05, ** *P* < 0.01, ***P < 0.001, ****P < 0.0001.

Since multimers in whole cell lysate and intracellular aggregated condensates were consistently found in our transfections, we next aimed to look into more details if GJB1-WT and mutants induce a distinct pattern of aggregates and employed sequential extraction using buffers containing different detergents for protein solubility. As illustrated in Figure 4A, NP40 buffer and SDS buffer were sequentially applied, and total extract, NP40 lysate, and SDS lysate were resolute. All spectra of signals were subgrouped into three species: aggregates, high-molecular-weight (HMW) multimers, and monomers and truncations. In the total extracts, compared to the WT, all the mutants including the truncated mutation showed a significant increase in all species, with truncation showing most aggregates (3.4-fold) and F31S showing most HMW multimers (4.0-fold) (Figures 4B,C). In NP40-soluble fractions, all trends were highly similar to what was found in the total extract (Figures 4D,E). In SDS-soluble fractions, compared to WT, F31S and R220Pfs^*^23 showed a 1.6- to 1.9-fold increase in aggregates, respectively, and all mutants indicated a 2.1- to 3.5-fold increase in HMW multimers. Surprisingly, R220Pfs^*^23 mutation showed upto 11.4-fold increased monomers, much >3.7- to 4.8-fold elevations in the F31S and W44G groups in contrast to the WT (Figures 4F,G). These findings again confirmed that all the mutations investigated in the study lead to enhanced intracellular accumulation of GJB1 aggregates, although at slightly varying degrees.

### Subcellular distribution of novel GJB1 variants

**Figure 5:**
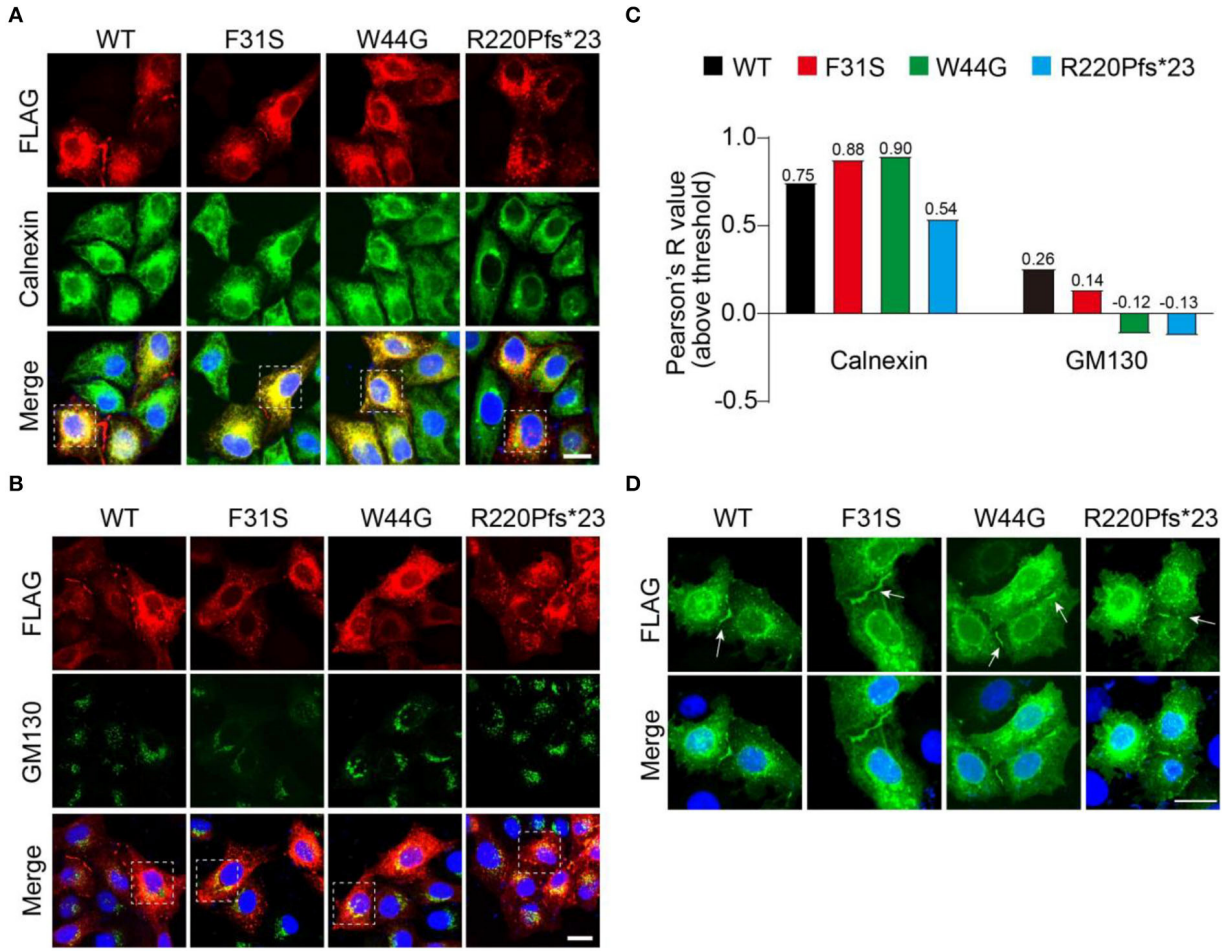
Accumulation of GJB1 aggregates in endoplasmic reticulum. **(A)** Immunofluorescence staining of FLAG-tagged GJB1 variants and endoplasmic reticulum maker protein Calnexin in transfected HeLa cells. **(B)** Co-staining of GJB1-FLAG and Golgi maker protein GM130 in transfected HeLa cells. **(C)** Colocalization analysis of GJB1-FLAG with Calnexin or GM130 in highlighted cells in A and B. **(D)** Labeling GJB1-FLAG in the transfected cell shows clear GJ plaque structures in all groups. Blue indicates DAPI-labeled nuclei. Arrows indicate GJ plaques. Scale bar, 20 μm.

After confirming the existence of all GJB1 variants including the WT, we wondered in which department these aggregates were formed during the protein processing. To address that, we labeled transfected GJB1-FLAG with endoplasmic reticulum (ER) marker Calnexin and Golgi marker GM130. Strong colocalization of GJB1 with Calnexin rather than GM130 was observed (Figures 5A,B), which was confirmed by colocalization analysis. The analysis also suggested that R220Pfs^*^23 aggregates showed the least localization in the ER or Golgi compared to other mutations (Figure 5C). GJ-plaque structures were observed in cells from all groups, confirming the fundamental role of GJB1 in forming gap junctions. However, R220Pfs^*^23 mutant shows much shorter and thinner GJ-plaque, indicative of possible impairment (Figure 5D).

### Aggregated GJB1 are ubiquitinated

**Figure 6:**
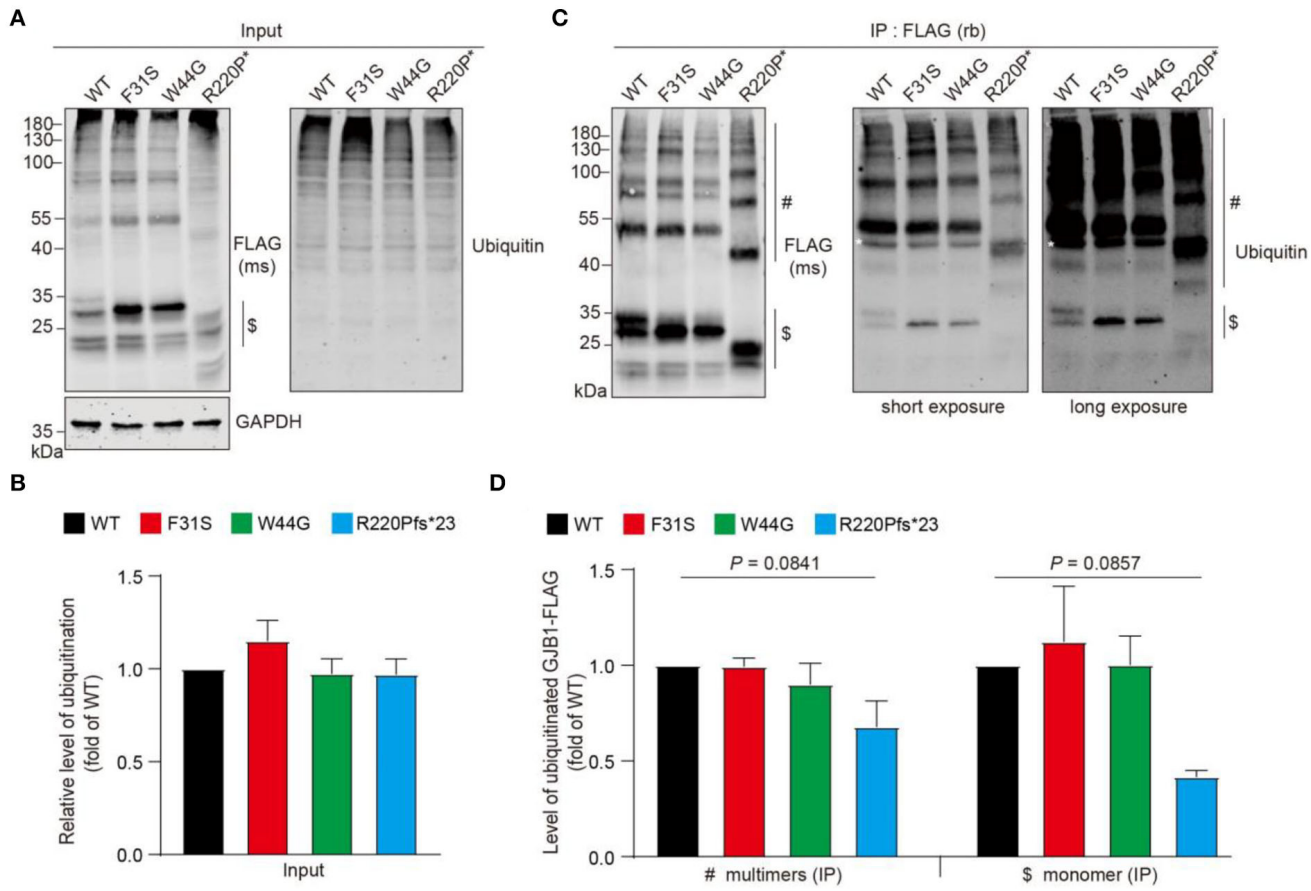
Ubiquitination assessment of GJB1 species in transfected HeLa cells. **(A)** GJB1-FLAG variants expressing HeLa cells were lysed as immunoprecipitation input and probed with anti-GJB1 antibody. Ubiquitinated proteins were detected with a pan anti-ubiquitin antibody. $ indicates GJB1 monomers. **(B)** Quantification of ubiquitination levels in input lysate (*n* = 4 per group). No significance was observed. **(C)** Cell lysates in A were immunoprecipitated (IP) with an anti-FLAG antibody raised in rabbit (rb) and then probed with an anti-FLAG antibody raised in mouse (ms) and an anti-ubiquitin antibody. White * in the blots indicates a heavy chain of the antibody. $ indicates GJB1 monomers and # indicate multimer species. **(D)** Quantification of ubiquitinated GJB1-FLAG was performed by normalizing band density of ubiquitin signal to GJB1-FLAG signal in corresponding samples (*n* = 3 per group).

Protein aggregation is often tightly associated with its state of ubiquitination, since dysregulation of ubiquitin-proteasome system-directed protein degradation may always account for persistent aggregation. We then wondered if these aggregates are modified with ubiquitin. Anti-FLAG probing in the whole cell lysate showed a similar pattern described previously, including the HMW multimers and aggregates. Pan-ubiquitin antibody probing in the whole cell lysate detected a typical smeared signal at the top of the lanes (Figure 6A), and quantification showed no change in total ubiquitinated protein levels between groups (Figure 6B). Immunoprecipitation suggested that GJB1-FLAG was virtually enriched in the immunoprecipitates with apparent GJB1-formed multimer ladder in lysate from all groups. With two degrees of exposure, ubiquitinated GJB1 was clearly observed, at the size of monomers and also higher sizes for the multimers (Figure 6C). Quantification of two groups of species suggested that the relative level of ubiquitinated GJB1-FLAG was not altered by F31S or W44G mutation, whereas the truncated GJB1 lysate presented less ubiquitination than the WT close to a significant difference (Figure 6D).

### GJB1 mutants trigger stress granule formation and impaired cell proliferation

**Figure 7:**
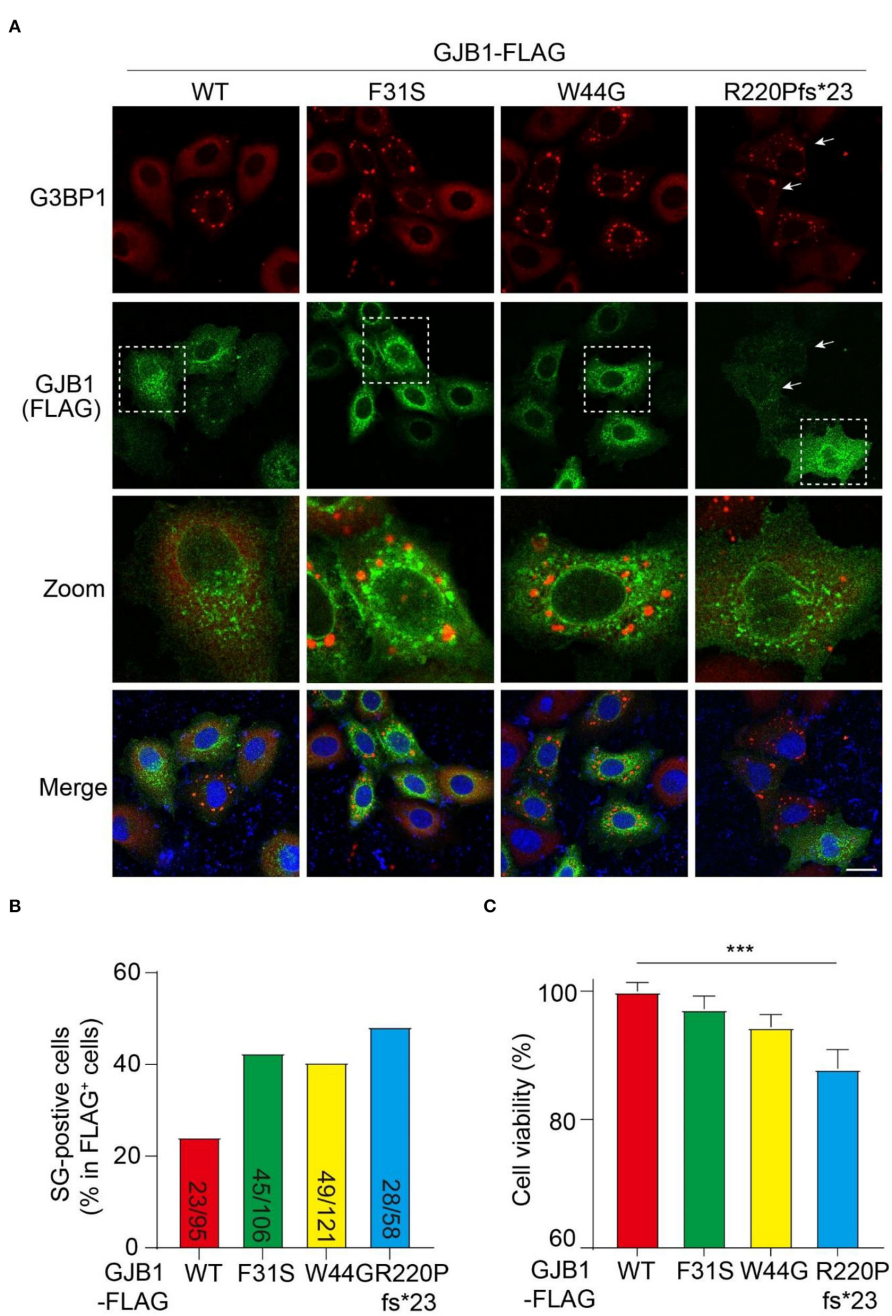
GJB1 mutations induce more stress granule formation and impaired cell activity. **(A)** Immunofluorescence staining of FLAG-tagged GJB1 variants within transfected HeLa cells. Red, anti-G3BP1 staining; Green, anti-FLAG staining; Blue, DAPI-labeled nuclei. Dashed boxes were zoomed in the panel below. Arrows indicate cells with obvious G3BP1 puncta but a small amount of GJB1. **(B)** Number of SG-positive cells and FLAG-positive cells were blindly counted from individual immunofluorescent images and were shown as indicated. **(C)** Cell activity of GJB1-overexpressing HeLa cells was determined by CCK-8 assay. ***P < 0.0001. Scale bar, 20 μm.

Persistent accumulation of aberrant protein aggregates is believed to cause cell stress and ultimate cell dysfunction. Therefore, we examined if cell stress is induced by mutants by probing the cells with G3BP1, a marker protein of SG which is a dynamic and reversible cytoplasmic assembly formed in eukaryotic cells in response to stress. In all GJB1-overexpressing cells, GJB1 appeared aggregates-like distribution similar to that described earlier. The size of these G3BP1-labeled SGs was greater than GJB1-positive aggregates, and they were not colocalized at all. Interestingly, most GJB1 WT-expressing cells were negative for G3BP1, whereas the majority of cells with F31S and W44G mutants exhibited extensively generated SGs. Moreover, truncated GJB1 showed a similar number of SG-positive cells vs. the other mutants (Figure 7A). Quantitative results suggested WT GJB1-FLAG overexpression was caused by SG formation in 24% of the cells while an almost two-fold increased number (40–48%) of SG-positive cells were found in mutants-expressing cells (Figure 7B). To further assess the functional consequences of GJB1 mutants, we examined the cell viability and cytotoxicity using the CCK-8 assay. About 24 h after transfection, cell viability in R220Pfs^*^23 mutant transfected cells was significantly declined than that in the WT (*P* < 0.001), whereas both F31S and W44G transfection had a mild tendency of attenuated cell proliferation or viability (Figure 7C).

## Discussion

Using targeted exome-sequencing, we identified two known *GJB1* mutations (p.Y157H and p.R220Pfs^*^23) and two novel pathogenic variants in *GJB1* (p.F31S and p.W44G) among four CMT families in central-southern China. F31S and W44G were first found in our cohort, expanding the mutation spectrum in GJB1-CMT1X. Both variations of Y157H and R220Pfs^*^23 have been previously reported, whereas only the Y157H mutation has been briefly explored functionally *in vitro*. None of the mutations in the EC2 domain (TM3-TM4), the hotspot in Chinese GJB1-CMT1X patients, was found in the present study. Here, we explored the functional consequences of corresponding mutations in cultured cells and discovered that mutations in GJB1 drive its self-aggregation and SG formation in the cytosol, which may further contribute to neuropathology in CMT.

### Rare CNS manifestation caused by frameshift mutation

*GJB1* mutations may lead to reversible PNS phenotype in CMT1X as well as CNS abnormality. The CNS manifestations of CMT1X primarily include episodic limbs or facial weakness, facial or limb numbness, dysarthria or dysphagia, ataxia, spasticity, hyperreflexia, and cognitive impairment (Niu et al., 2019; Tian et al., 2021). All four probands in the present study shared a similar phenotype of early-onset, slowly progressive distal muscle weakness, decreased or absent tendon reflex, and pes cavus. Interestingly, the proband 3 carrying the *GJB1*p.R220Pfs^*^23 mutation had neurologic manifestations that are involved not only in the PNS but also in the CNS. In a large Chinese cohort of CMT patients, Liu et al. reported a male patient with the same mutation as proband 3, with pure signs of peripheral neuropathy (Liu et al., 2020). This indicates that *GJB1* mutations might give rise to a wild phenotypic spectrum, making diagnosis challenging due to phenotypic variability. Moreover, CNS abnormalities were found in proband 3 but were not present in other affected individuals within the family, showing high intrafamilial phenotypic variability.

### GJB1 aggregation and GJ-plaque formation

*GJB1*-encoding GJB1 protein is primarily expressed in both Schwann cells and oligodendrocytes and is a GJ-forming protein that mediated intercellular communication. Six connexins come together to form a hemichannel arranged around a central pore, and two apposed hemichannels dock together to form gap junction channels (Abrams, 2019), which are located in the cell membrane forming GJ plaques (Kleopa et al., 2012). The majority of the mutations are expected to cause loss of GJB1 function in myelinating Schwann cells (Shy et al., 2007). Growing evidence suggests that failure to form plaques or functional GJ channels and inappropriate trafficking of GJB1 were involved in the pathogenesis of CMT1X (Gong et al., 2013; Abrams et al., 2017; Carrer et al., 2018). In agreement with previous reports, we revealed that the GJ plaques formed by GJB1 mutants were indistinguishable from WT, with possible impairment in the frameshift group (Figure 5D) (Kleopa et al., 2002).

In our study, HMW multimers and aggregated forms of WT-GJB1 and mutants are consistently revealed by immunostaining, immunoblotting, and immunoprecipitation, supported by evidence showing aggregation is also initiated when GJB1 is low expressed. Sequential fractionation further demonstrated apparently elevated GJB1 aggregation in all mutants in contrast to WT (Figure 4). Although heat-denaturation may affect the presentation of composition of those species, the existence of SDS-resistant GJB1 multimers with aggregates is apparent, and exaggerated levels of all species in mutant groups are also valid.

Mutation-directed GJB1 aggregation may deplete the protein pool in the cell, on one hand, and disrupt the functional formation of GJ channels, on the other hand, resulting in cell stress. Whether mutation-directed GJB1 aggregations are anti-toxic or pro-toxic in general is still unknown. Furthermore, immunoprecipitation indicates that aggregates are ubiquitinated, although at comparable levels, suggesting that the ubiquitination-dependent degradation system might not be directly linked to GJB1 aggregation and self-aggregation of GJB1 is responsible for the elevated insolubility. GJB1 mutants appear to be concentrated in an intracellular compartment such as the ER, the Golgi apparatus, or the cytoplasm, because of impaired trafficking of GJB1 to the plasma membrane (Bortolozzi, 2018; Guo et al., 2021). Strong colocalization of GJB1 in the ER rather than the Golgi has also been observed, and the aggregates of frameshift mutation seem to have the least ER localization (Figures 5A,B), possibly due to its unique property of high-degree misfolding presented in the SDS-soluble fraction.

### Association of GJB1 aggregation and SGs

The assembly of SGs, transient membraneless organelles, is a well-known cellular strategy for reducing stress-related damage and promoting cell survival. Mutations causing misfolded proteins might enhance SGs formation and/or stress granule formation that might stimulate protein misfolding. G3BP1 is a tunable switch that triggers phase separation to assemble SGs. G3BP1 inhibits ubiquitinated protein aggregations induced by p62 and USP10 (Anisimov et al., 2019), which may explain the G3BP1-positive SGs formed in mutated GJB1-expressing cells in which GJB1 aggregation was not ubiquitinated. Overexpression of both WT and mutant GJB1 lead to the formation of SGs, with significantly increased SGs and decreased cell viability by the mutants. It is possible that the mild presentation of SGs in the WT group is due to the stress from the redundant expression of exogenous expression. Interestingly, the SGs and intracellular aggregation are not colocalized, suggesting GJB1 itself is excluded from the SGs. Mutations in GJB1 lead to misfolded GJB1 accumulation and SGs formation, which may in turn stimulate the misfolding of GJB1, forming a vicious feedback cycle and promoting pathogenesis.

### Frameshift mutation drives most severe aggregation and cell stress

Among these *GJB1* variants, despite the peripheral neuropathy found in all probands, the carrier of frameshift mutation exhibits rare CNS abnormalities. We show distinct properties of this frameshift mutation from other missense mutations in our cohort. As indicated by immunoblotting and immunostaining, frameshift-mutated GJB1 presents the highest levels of aggregates in the total extract and highest levels of HMW multimers and aggregates in the SDS-soluble fractions, suggesting the unique tendency of this mutation prone to fibrillation. SG-labeling also reflects that cells are more sensitive to frameshift-induced aggregates than the other two mutations. More importantly, these aggregates showed the greatest cytotoxicity as indicated by the viability assay. These findings together suggest that cells may be less intolerant to frameshift-directed aggregation and cytotoxicity, which may give hints that only frameshift-mutated GJB1 causes CNS abnormalities since CNS cells sense more stress from this mutation.

### Limitations

Given that more than 450 GJB1 mutations have been reported in CMT1X, we were not able to compare the functional consequences of all mutations to our newly found mutations, also due to a lack of biochemical validation on previously discovered mutations. We propose that similar aggregation and SGs formation with other mutations are largely possible. Even though we employed a mammalian cell line in our study, relative cell types such as Schwann cells and oligodendrocytes would be more favorable in understanding how mutations of GJB1 contribute to CMT1X phenotypes in PNS and CNS. Artifacts from overexpression in the cultured cell are not excluded. A functional alternation such as electrophysiological analysis on gap junction channels was not determined. Pathogenic post-translational modifications in GJB1 and the reason why WT GJB1 forms visible aggregations remains unclear.

## Conclusion

**Figure 8:**
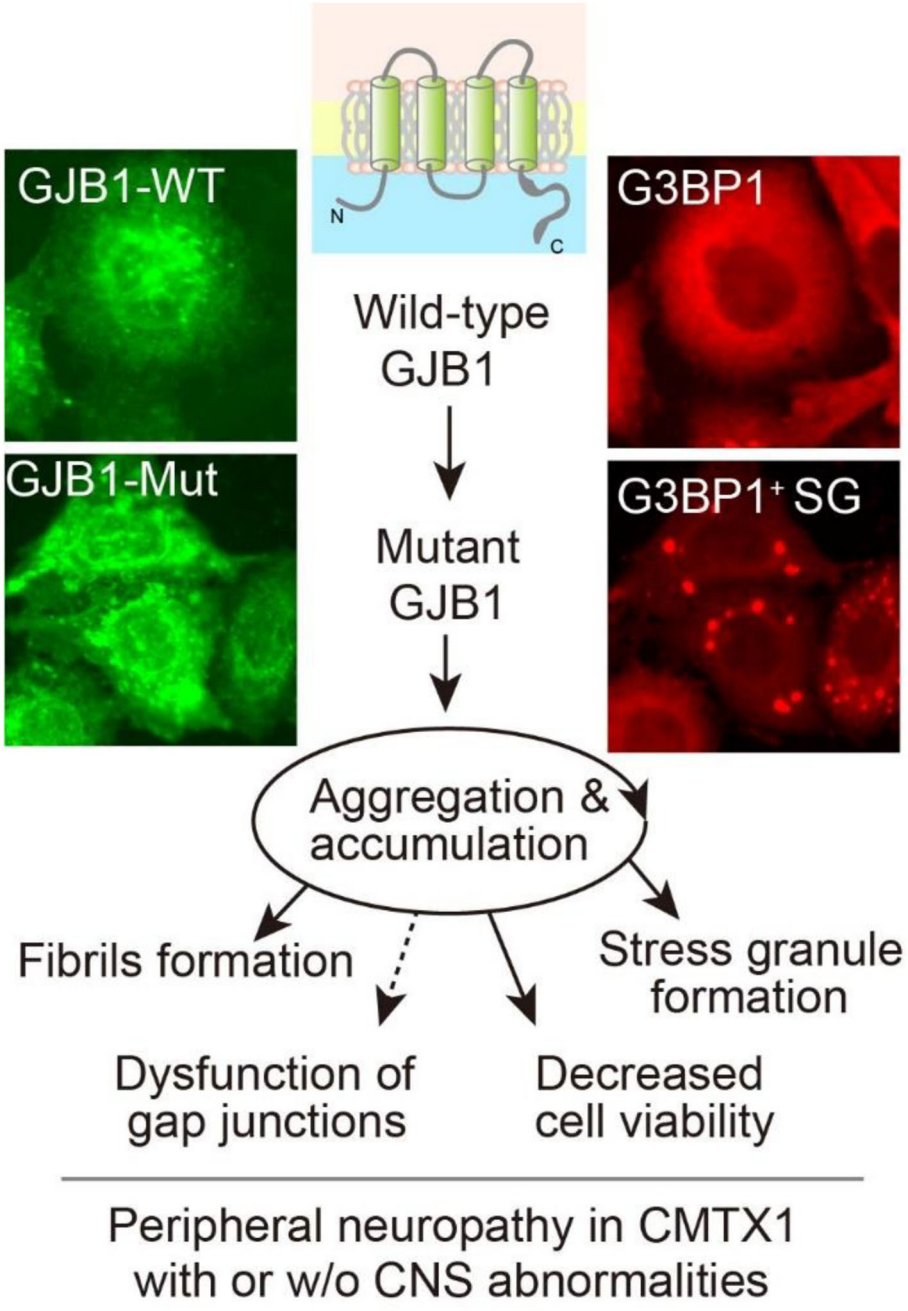
Graphic working hypothesis of *GJB1* mutation-induced CMT. As a gap junction protein, GJB1 normally spans the cell membrane to form gap junction channels. Ectopic WT GJB1 expression leads to its aggregation at a low extent, whereas mutations in GJB1 trigger a vicious circle *via* aggregation and accumulation of GJB1 species, which further causes intracellular stress granules formation and compromised viability. All the above evidence reflects CMT pathology in the peripheral system and possibly in CNS when mutation results in severe cell damage.

In summary, we identified four likely pathogenic variants, two of which are novel, in *GJB1* among four Chinese CMT families, which expand the mutational spectrum of the GJB1-related CMT disease. *In vitro* analysis further confirmed that GJB1 mutants altered protein expression and showed increased properties prone to aggregate intracellularly, leading to possible disruption of gap junction channels, accelerated stress granules formation, and cytotoxicity generation (Figure 8). Despite the phenotypic variability in GJB1-CMT1X, our findings will help us understand the pathomechanism of the disease and potential genotype-phenotype correlations.

## Data availability statement

The original contributions presented in the study are included in the article/supplementary material, further inquiries can be directed to the corresponding authors.

## Ethics statement

The studies involving human participants were reviewed and approved by Wuhan Union Hospital, Huazhong University of Science and Technology. The patients/participants provided their written informed consent to participate in this study.

## Author contributions

FC, JX, and YoW: methodology, software, formal analysis, data curation, and writing—original draft and visualization. YL and YaW: software, data curation, and writing—review and editing. CL and ZL: conceptualization, methodology, writing—review and editing, supervision, and funding acquisition. All authors contributed to the article and approved the submitted version.

## Funding

This work was supported by the grants from the National Natural Science Foundation of China (31900692) to CL and grants from the National Natural Science Foundation of China (82101504) to ZL.

## Conflict of interest

The authors declare that the research was conducted in the absence of any commercial or financial relationships that could be construed as a potential conflict of interest.

## Publisher's note

All claims expressed in this article are solely those of the authors and do not necessarily represent those of their affiliated organizations, or those of the publisher, the editors and the reviewers. Any product that may be evaluated in this article, or claim that may be made by its manufacturer, is not guaranteed or endorsed by the publisher.
