# Corrigendum: Novel mutations in GJB1 trigger intracellular aggregation and stress granule formation in X-linked Charcot-Marie-Tooth Disease

**DOI:** 10.3389/fnins.2023.1282954

**Published:** 2023-09-12

**Authors:** Fan Chu, Jiaming Xu, Yong Wang, Yingjie Li, Yaling Wang, Zhijun Liu, Chuanzhou Li

**Affiliations:** ^1^Department of Medical Genetics, School of Basic Medicine, Tongji Medical College, Huazhong University of Science and Technology, Wuhan, China; ^2^Department of Neurology, Union Hospital, Tongji Medical College, Huazhong University of Science and Technology, Wuhan, China

**Keywords:** GJB1, gap junction, aggregation, stress granule, Charcot-Marie-Tooth

In the published article, there was an error in “Table 1” as published. We are correcting the error in the original Table 1, i.e., the p.R164Q mutation we reported should be corrected as p.Y157H. The corrected “Table 1” and its caption “The clinical presentations and genetic analysis of four CMT probands” appear below.

**Table 1:** The clinical presentations and genetic analysis of four CMT probands.

**Characteristics**	**Proband 1**	**Proband 2**	**Proband 3**	**Proband 4**
Age, years	29	23	25	28
Sex	Male	Male	Male	Male
Age at onset, years	23	22	Early childhood	15
Initial symptoms	Weakness and atrophy in hands	Weakness of right hands	Running difficulty	Right lower limbs weakness
Pathogenic variants in *GJB1*	p.W44G	p.F31S	p.R220Pfs^*^23	p.Y157H
Muscle atrophy UL/LL	+/++	++/+	++/++	++/+++
Deep tendon reflexes	Decreased	Decreased	Disappeared	Disappeared
Pes cavus	Yes	Yes	Yes	Yes
Sensory loss	No	Yes	No	Yes
Brain MRI	Normal	Normal	Hyperintensity in the bilateral corona radiate on T2WI	Normal
Other features	–	–	Stroke-like episodes	–
CMAP in median/ulnar nerves, mv	1.0/3.5	0.3/0.7	1.0/3.1	NA
MCV in median/ulnar nerves, m/s	29.9/32.4	25.3/26.8	28.6/31.1	NA
SNAP in median/ulnar nerves, uv	5.5/7.0	4.0/4.5	4.6/6.3	NA
SCV in median/ulnar nerves, m/s	38.2/37.3	32.1/33.4	35.4/36.5	NA

UL, upper limbs; LL, low limbs; +, mild; ++, moderate; +++, severe; CMAP, compound motor action potential; MCV, motor conduction velocity; SNAP, sensory nerve action potential; SCV, sensory conduction velocity; NA, not available.

In the published article, there was an error. The mutation p.R164Q we reported should be corrected as p.Y157H. A correction has been made to “Pages 1, 4, 6, and 12”.

This sentence previously stated:

Page 1. Abstract

“Using targeted exome-sequencing, we investigated four CMT families from central-southern China and identified two novel missense variants (p.F31S and p.W44G) and two previously reported variants (p.R220Pfs^*^23 and p.R164Q) of GJB1.”

Page 4. Results section, paragraph 1

“After tering and validation by Sanger sequencing, four probable pathogenic variants, including two variants previously reported as pathogenfilic variants (c.469T>C, p.R164Q, Li et al., 2016 and c.657dupC, p.R220Pfs^*^23) (Lu et al., 2017) and two novel variants (c.130T>C, p.F31S and c.92T>C, p.W44G), were identified in four CMT families (Figure 1).”

Page 6, right column, paragraph 1

“The proband 4 (III:2) was a 28-year-old male, carrying the p.R164Q variant in *GJB1*, who presented with right lower limb weakness at the age of 15.”

Page 6, right column, paragraph 2

“Since functional alternation by R164Q variation has been briefly explored previously (Li et al., 2016), we have not included this mutation in our study.”

Page 12, Discussion section, paragraph 1

“Using targeted exome-sequencing, we identified two known *GJB1* mutations (p.R164Q and p.R220Pfs^*^23) and two novel pathogenic variants in *GJB1* (p.F31S and p.W44G) among four CMT families in central-southern China. F31S and W44G were first found in our cohort, expanding the mutation spectrum in GJB1-CMT1X.”

Page 12, Discussion section, paragraph 1

“Both variations of R164Q and R220Pfs^*^23 have been previously reported, whereas only the R164Q mutation has been briefly explored functionally *in vitro*.”

The corrected sentence appears below:

Page 1. **Abstract**

“Using targeted exome-sequencing, we investigated four CMT families from central-southern China and identified two novel missense variants (p.F31S and p.W44G) and two previously reported variants (p.R220Pfs^*^23 and p.Y157H) of GJB1.”

Page 4. **Results** section, paragraph 1

“After filtering and validation by Sanger sequencing, four probable pathogenic variants, including two variants previously reported as pathogenic variants (c.469T>C, p.Y157H, (Li et al., 2016) and c.657dupC, p.R220Pfs^*^23) (Lu et al., 2017) and two novel variants (c.130T>C, p.F31S and c.92T>C, p.W44G), were identified in four CMT families (Figure 1).”

Page 6, right column, paragraph 1

“The proband 4 (III:2) was a 28-year-old male, carrying the p.Y157H variant in *GJB1*, who presented with right lower limb weakness at the age of 15.”

Page 6, right column, paragraph 2

“Since functional alternation by Y157H variation has been briefly explored previously (Li et al., 2016), we have not included this mutation in our study.”

Page 12, **Discussion** section, paragraph 1

“Using targeted exome-sequencing, we identified two known *GJB1* mutations (p.Y157H and p.R220Pfs^*^23) and two novel pathogenic variants in *GJB1* (p.F31S and p.W44G) among four CMT families in central-southern China. F31S and W44G were first found in our cohort, expanding the mutation spectrum in GJB1-CMT1X.”

Page 12, **Discussion** section, paragraph 1

“Both variations of Y157H and R220Pfs^*^23 have been previously reported, whereas only the Y157H mutation has been briefly explored functionally *in vitro*.”

The authors apologize for this error and state that this does not change the scientific conclusions of the article in any way. The original article has been updated.
